# Effect of high-dose intravenous ascorbic acid on microcirculation and endothelial glycocalyx during sepsis and septic shock: a double-blind, randomized, placebo-controlled study

**DOI:** 10.1186/s12871-023-02265-z

**Published:** 2023-09-12

**Authors:** Egle Belousoviene, Zivile Pranskuniene, Egle Vaitkaitiene, Vidas Pilvinis, Andrius Pranskunas

**Affiliations:** 1 Department of Intensive Care Medicine, Lithuanian University of Health Sciences, Eiveniu g. 2, Kaunas, LT-50161 Lithuania; 2 Department of Drug Technology and Social Pharmacy, Lithuanian University of Health Sciences, Sukileliu pr.13, Kaunas, LT-50162 Lithuania; 3 Institute of Pharmaceutical Technologies, Lithuanian University of Health Sciences, Sukileliu pr.13, Kaunas, LT-50162 Lithuania; 4 Department of Disaster Medicine and Health Research Institute, Lithuanian University of Health Sciences, Eiveniu g. 4, Kaunas, LT-50161 Lithuania

**Keywords:** Microcirculation, Ascorbic acid, Endothelial glycocalyx, Antioxidants

## Abstract

Previous studies indicate supplemental vitamin C improves microcirculation and reduces glycocalyx shedding in septic animals. Our randomized, double-blind, placebo-controlled trial aimed to investigate whether a high dose of intravenous ascorbic acid (AA) might improve microcirculation and affect glycocalyx in septic patients. In our study, 23 septic patients were supplemented with a high dose (50 mg/kg every 6 h) of intravenous AA or placebo for 96 h. Sublingual microcirculation was examined using a handheld Cytocam-incident dark field (IDF) video microscope. A sidestream dark field video microscope (SDF), connected to the GlycoCheck software (GlycoCheck ICU®; Maastricht University Medical Center, Maastricht, the Netherlands), was employed to observe glycocalyx. We found a significantly higher proportion of perfused small vessels (PPV) 6 h after the beginning of the trial in the experimental group compared with placebo. As an indicator of glycocalyx thickness, the perfused boundary region was lower in capillaries of the 5–9 μm diameter in the AA group than placebo after the first dose of AA. Our data suggest that high-dose parenteral AA tends to improve microcirculation and glycocalyx in the early period of septic shock. The study was retrospectively registered in the clinicaltrials.gov database on 26/02/2021 (registration number NCT04773717).

**Supplementary Information:**

The online version contains supplementary material available at 10.1186/s12871-023-02265-z.

## Introduction

Sepsis and septic shock remain the leading causes of death in intensive care units worldwide [1]. Evidence suggests oxidative stress and microcirculatory disturbances are the driving mechanisms of sepsis [2, 3]. During sepsis, released cytokines activate macrophages, neutrophils, and platelets and promote endothelial dysfunction. Reactive oxygen species (ROS) generation is increased, oxidative phosphorylation and ATP production in mitochondria are disturbed, and there is a reduction in organ perfusion. Consequently, the reduction in perfused capillary density results in tissue hypoxia [2, 4]. It has been demonstrated that the severity of microcirculatory alterations during sepsis is linked with mortality [5]. In addition, microvascular alterations improved over 24 h after the onset of shock in response to therapy in survivors but not in non-survivors [6]. For practical reasons, changes in human microcirculation during sepsis are predominantly evaluated in the sublingual region using handheld microscopes, including an IDF and SDF imaging.

Signs of impaired tissue perfusion during sepsis persist despite apparent successful restoration of systemic hemodynamics [4, 7]. Some hypotheses link this hemodynamic incoherence to endothelial dysfunction, imbalance between endogenous vasoconstrictors and vasodilators, glycocalyx damage, endogenous antioxidants depletion, and increased oxidant production [4, 8].

The most abundant endogenous antioxidant is ascorbate, the reduced form of vitamin C [2]. Humans have lost the ability to synthesize it endogenously in the course of evolution due to random mutations in the L-gulono-γ-lactone gene. This loss did not influence survival because there is sufficient vitamin C in the human diet [9]. However, the level of ascorbic acid decreases in critical illnesses such as sepsis [10, 11]. Ascorbic acid levels are significantly lower at the early stages of the disease, approaching those observed in scurvy patients, and they are inversely correlated with multiorgan dysfunction measures [2].

Previously published studies in animals indicate that supplemental vitamin C attenuates systemic inflammation and vascular injury, reduces glycocalyx shedding [9], corrects sepsis-induced coagulopathy [11], and improves microcirculation [2, 12]. However, there is a lack of data on ascorbic acid’s effect on microcirculation in humans. Our randomized, double-blind, placebo-controlled trial aimed to investigate whether a high dose of supplemental intravenous ascorbic acid might improve microcirculatory parameters in patients suffering from sepsis.

## Materials and methods

The Kaunas Regional Biomedical Research Ethics Committee approved the study (number of approval BE-2-5). The study adopted a randomized, double-blind, placebo-controlled format. Written informed consent was obtained from patients or their legal representative following national regulations. Investigations were carried out according to the principles of the Declaration of Helsinki. The study was retrospectively registered in the clinicaltrials.gov database (registration number NCT 04773717).

### Participants

**Fig. 1:**
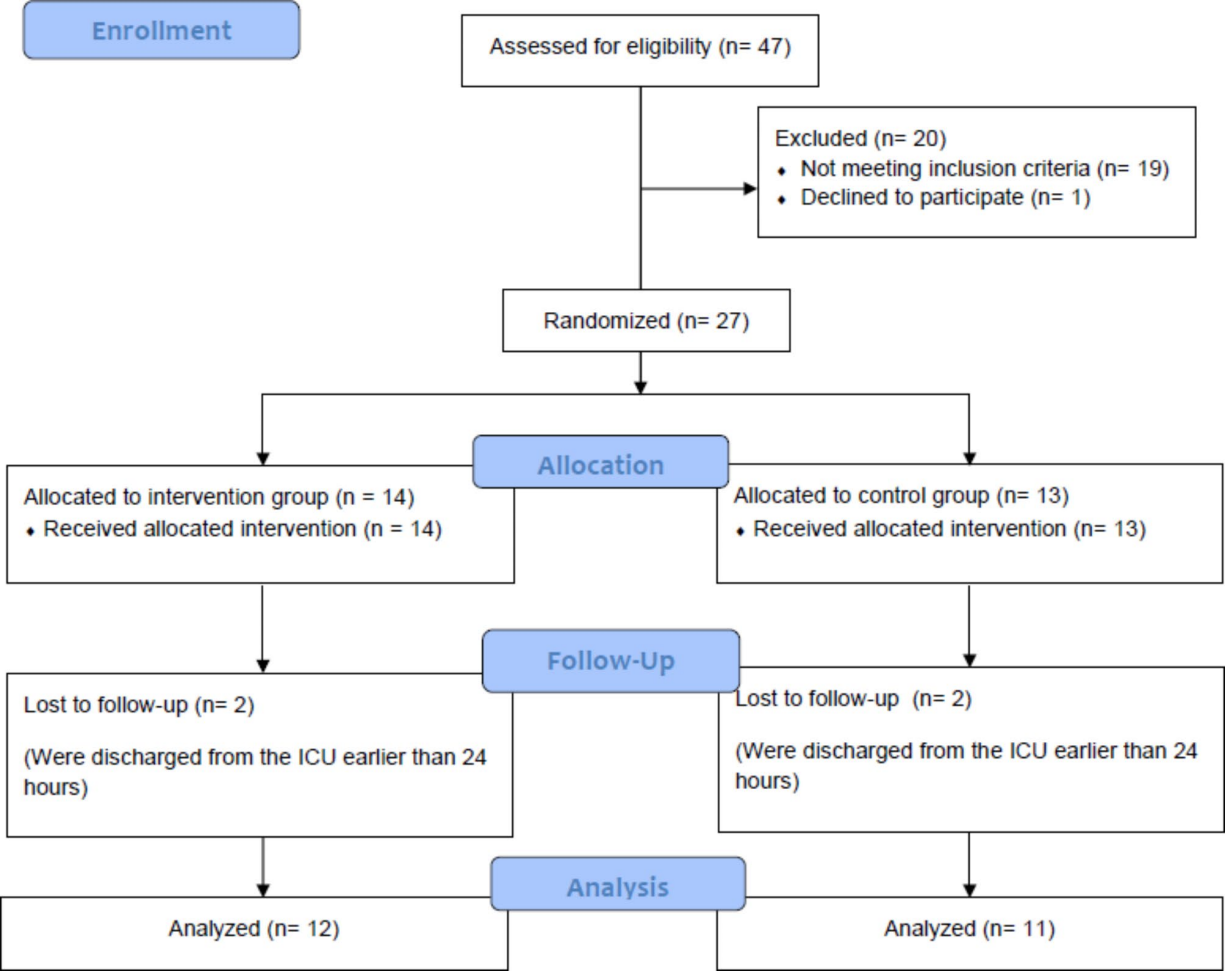
Flow diagram of the study

Twenty-three adult patients with sepsis and septic shock were enrolled in the study within 24 h following admission to the Central Department of Intensive Care in Lithuanian University of Health Sciences Hospital Kaunas Clinics during a period between 2019 January to 2021 January (Fig. 1).

Following the third international consensus definition, septic shock was defined as sepsis requiring vasopressor therapy to elevate MAP > 65mmHg and lactate concentration greater than 2 mmol/L despite adequate fluid resuscitation. Sepsis was diagnosed in the presence of suspected or documented infection and an acute increase of ≥ 2 points in the Sequential Organ Failure Assessment (SOFA) score [13].

Patients were excluded if consent was impossible to obtain; if they were younger than 18 years old; if pregnant or breastfeeding; if moribund and not expected to survive 24 h; if there was a known history of kidney stone, glucose-6-phosphate deficiency, hemochromatosis, or solid organ transplantation; as well as and if the sublingual mucosa was damaged.

### Study protocol

The study groups in this trial received either placebo (NaCl 0.9% and a clinically insignificant amount of riboflavin as colorant) or ascorbic acid. Participants were assigned to the placebo or ascorbic acid group in a 1:1 ratio using the online tool Research Randomizer [14].

All patients had a central venous catheter and an arterial line and were resuscitated following international sepsis management guidelines to optimize their hemodynamics [1]. Additionally, either 50 mg/kg of ascorbic acid or placebo was administered over 30 min into a central line every 6 h for 96 h.

The ascorbic acid manufacturer helped to ensure the blind format of the study. The 50 mL amber vials containing either ascorbic acid (concentration of 150 mg/mL) or placebo were supplied in separate kits for every patient. They were coded according to numbers, and unblinding occurred only after patient recruitment was complete. Placebo or ascorbic acid was diluted with 5% dextrose solution immediately before infusion into 50 mL amber-colored syringes, and light-protected infusion systems were used.

Ascorbic acid and placebo vials were stored at 2–8 °C before use. The first dose was infused within 4 h after randomization. Study drug administration was stopped following the infusion of the last dose (96 h), ICU discharge, or death.

Systemic hemodynamic variables, sublingual microcirculation, glycocalyx parameters, and arterial blood gas analysis results were evaluated at baseline and then within 30 min after the cessation of the first ascorbic acid or placebo infusion, and at 6, 12, 24, 48, 72, and 96 h from the beginning of the study. The Acute Physiology and Chronic Health Evaluation II (APACHE II) score was calculated over the first 24 h following ICU admission. ΔSOFA score was evaluated within 96 h. Standard laboratory tests were performed daily, as usual, in critically ill patients.

Blood samples to estimate interleukin-6 (IL6) and interleukin-10 (IL10) levels were drawn before the first study drug infusion and after 24 h. ICU mortality was registered.

### Evaluating microcirculation

Video images of the sublingual microcirculation were obtained using a handheld Cytocam IDF video microscope (Braedius Medical, Huizen, the Netherlands). This tool is developed to register the microcirculation in organ surfaces. The IDF imaging principle is based on the absorbance, by hemoglobin, of the green light (wavelength: 530 nm) emitted by the microscope. Consequently, red blood cells are visualized as black or gray dots.

It is impossible to visualize the vessel walls using this device, and the capillaries are only visible when filled with red blood cells [15].

A recently published validation study demonstrated that Cytocam IDF imaging offers better image quality than SDF imaging [16].

After gently removing saliva with isotonic saline-drenched dressing, the microscope was applied to the sublingual mucosa while avoiding pressure artifacts, and image sequences from at least three sections were captured. Trained investigators used validated AVA v.3.2 software (AVA, MicroVision Medical BV, Amsterdam, the Netherlands) in the blinded analysis of video clips. The clips were arranged in a random order to avoid coupling. Expert recommendations [17, 18] were followed for determining the quality as well as for the analysis of recorded images.

Each image was divided into four equal quadrants, whereby the flow in each was quantified by eye (0, no flow; 1, intermittent flow; 2, sluggish flow; 3, continuous flow) for each vessel diameter cohort (small, 10–20 μm; medium, 21–50 μm; large, 51–100 μm). The microvascular flow index (MFI) was calculated as the sum of each quadrant score divided by the number of quadrants in which the vessel type was visible. The final MFI was an average of at least 12 quadrants (three regions, four quadrants per region) derived from the overall flow impressions for all vessels within a particular range of diameters in a given quadrant [19, 20]. The total vessel density (TVD) was calculated using the AVA software package for small vessels (primarily capillaries) and a cut-off diameter of < 20 μm. The proportion of perfused vessels (PPV) among small vessels was determined by dividing the length of the perfused small vessels by the total length of all small vessels. The perfused vessel density (PVD) of the small vessels was calculated by measuring the density of all perfused small vessels within the field of view (computed as the proportion of perfused vessels multiplied by the total vessel density). Flow heterogeneity index (FHI) for small vessels was calculated as the highest MFI minus the lowest MFI, divided by the mean MFI [17, 18].

### Evaluating glycocalyx

Visualizing human glycocalyx directly in vivo is highly challenging due to its fragility. The region where it is partially accessible to flowing red blood cells at its luminal side is called the perfused boundary region (PBR) [21]. It is measured as the distance from the median (P50) RBC column width to the (estimated) outer edge of the RBC-perfused lumen [22, 23]. Degradation of the glycocalyx results in deeper RBC penetration toward the endothelium and increased PBR [24].

An SDF video microscope attached to a glycocalyx measurement system (GlycoCheck ICU®; Maastricht University Medical Center, Maastricht, the Netherlands) was employed to assess sublingual microcirculation. Ten image sequences of forty frames were recorded in different areas, and PBR was automatically calculated. The RBC column was automatically measured in 3000 vascular segments. For each segment, 840 radial intensity profiles were captured to measure the RBC column width, and the PBR was automatically estimated. The vessel segments were classified into 1 μm wide diameter classes. PBR values were determined for each diameter class before calculating the average PBR over a set of diameters ranging from 5 to 25 μm. As described above, vessel diameters were categorized into groups of small (5–9 μm), medium (10–19 μm), and large (20–25 μm) to facilitate further analysis [22, 23].

### Statistics

The primary aim of our pilot study was to determine whether there are any differences in PPV, MFI, in septic patients depending on whether they received ascorbic acid vs. placebo. The sample size was in accordance with comparable previous studies [25, 26]. Eleven patients per group proved to be sufficient to demonstrate a change in MFI of 0.5 (standard deviation = 0.38) and change in PPV of 10% (standard deviation 7.9%) between two groups with a power of 80% and an alpha error of 0.05 [26].

A secondary aim was to determine the differences in PBR and systemic hemodynamics between groups of septic patients receiving either ascorbic acid or placebo. Data were analyzed with Statistical Package for Social Sciences (SPSS 22 for Windows, Chicago, USA). Due to the small sample size, data are presented as the median (25th–75th percentiles) and analyzed using non-parametric tests. Differences between groups were tested using a Mann–Whitney *U* test. Friedman’s test was conducted to assess changes in quantitative parameters over multiple time points in each group, followed by a Wilcoxon test to evaluate intragroup changes between two-time points. *p* < 0.05 was considered to indicate statistically significant differences. *Bonferroni correction* was used to reduce the chance of obtaining false-positive results (type I errors) when multiple pairwise comparisons were performed.

## Results

### Baseline characteristics

The study enrolled 23 of 27 patients who met the inclusion criteria. Four patients were excluded because their condition improved, resulting in their discharge from ICU earlier within 24 h from diagnosis of sepsis. Written informed consent was not obtained from one patient. The general demographic and clinical data of the studied patients are presented in Table 1. Patients did not differ significantly in baseline characteristics.

**Table 1:** Baseline characteristics of septic patients

Variables	AA group (n = 12)	Placebo group (n = 11)	p
Age (years)	66 (56–78)	60 (50–76)	0.391
Weight (kg)	84 (68–90)	76 (70–95)	0.967
Time to first dose (h)	15 (3–24)	10 (5–15)	0.596
MAP (mmHg)	80 (71–94)	80 (75–85)	1.000
HR (bpm)	125 (95–135)	108 (91–112)	0.133
Lactate level (mmol/L)	2.4 (1.3–4.7)	1.6 (1.4-3.0)	0.315
PBR_5 − 25_ (μm)	2.00 (1.76–2.44)	2.17 (1.97–2.33)	0.536
PBR_5 − 9_ (μm)	1.13 (1.01–1.25)	1.16 (1.09–1.29)	0.536
PBR_10 − 19_ (μm)	2.09 (1.81–2.55)	2.15 (1.98–2.42)	0.837
PBR_20 − 25_ (μm)	2.61 (2.45–3.21)	2.77 (2.48–2.99)	0.918
TVD (mm/mm^2^)	21.5 (19.6–24.3)	21.8 (18.8–23.1)	0.356
PVD (mm/mm^2^)	16.8 (15.3–20.8)	18.1 (14.4–21.5)	0.666
PPV (%)	81.8 (69.5–89.6)	83.4 (78.4–90.9)	0.605
MFI	1.54 (1.31–1.85)	1.83 (1.5–1.92)	0.297
FHI (AU)	0.25 (0.15–0.36)	0.28 (0.22–0.44)	0.500
Source of infection:			
Pneumonia n (%)	4 (33.3)	5 (45.5)	
Abdomen n (%)	7 (58.4)	1 (9.1)	
Urinary tract n (%)	1 (8.3)	3 (27.3)	
Other n (%)		2 (18.1)	
PEEP (cmH_2_O) FiO_2_ (%)	5 (5–8) 60 (40–70)	6 (5–8) 60 (50–65)	0.549 0.842
pH	7.33 (7.22–7.38)	7.24 (7.19–7.38)	0.661
pO_2_ (mmHg)	111 (80–138)	71 (58–121)	0.113
pCO_2_ (mmHg)	39 (34–59)	35 (31–42)	0.133
HCO_3_^−^ (mmol/L)	21 (17–25)	16 (14–21)	0.211
Hb (g/L)	117 (95–139)	106 (83–115)	0.243
CRP (mg/L)	214 (129–395)	277 (218–431)	0.497
Glucose level (mmol/L)	6.8 (5.5–9.8)	8.6 (6.9–12.8)	0.113

MAP, mean arterial pressure; HR, heart rate; PBR, perfused boundary region; TVD, total small vessel density; PVD, perfused small vessel density; PPV, proportion of perfused small vessels; MFI, microvascular flow index of small vessels; FHI, flow heterogeneity index; PEEP, positive end expiratory pressure; FiO2, inspired oxygen fraction; pO2,partial oxygen pressure; pCO2, partial carbon dioxide pressure, HCO3- , bicarbonate; Hb, hemoglobin; CRP, C reactive protein

Data are presented as median and 25th and 75th percentiles

Nine males (75%) and three females (25%) were randomly assigned to the experimental group. Six male patients (55%) and five female patients (45%) were assigned to the control group.The abdomen was the predominant source of infection (58.4%) in the experimental group, followed by pneumonia. Respiratory tract infections were dominant in the control group (45.5%), followed by 9.1% intraabdominal infections. Three patients in a placebo group had diabetes. There was no significant difference between the experimental and control groups regarding APACHE II (21 (13–24) and 17 (13–28), p = 0.838) and SOFA (10.0 (8.5–12.5) and 8.0 (7.0–9.0), p = 0.564) scores or initial doses of noradrenaline (0.20 (0.11–0.30) and 0.24 (0.20–0.35), p = 0.482), respectively. All patients required norepinephrine at the beginning of the trial. At inclusion all patients were under mechanical ventilation.

Systemic hemodynamic parameters did not differ significantly between groups at baseline or the end of the study. Vitamin C did not reduce norepinephrine dose compared with placebo. No significant differences were found in the levels of C reactive protein between the groups over the course of the study and the levels of interleukin-6 and interleukin-10 at the baseline and after 24 h from the beginning of the study.

No significant differences were found between the groups among the ΔSOFA96 in the AA group compared with placebo: 0 (0–4) vs. 2 (0–4), p = 0.356.

ICU mortality was 58.3% in the experimental group vs. 54.5% in the control group (p = 0.849).

### Evaluation of microcirculation

**Fig. 2:**
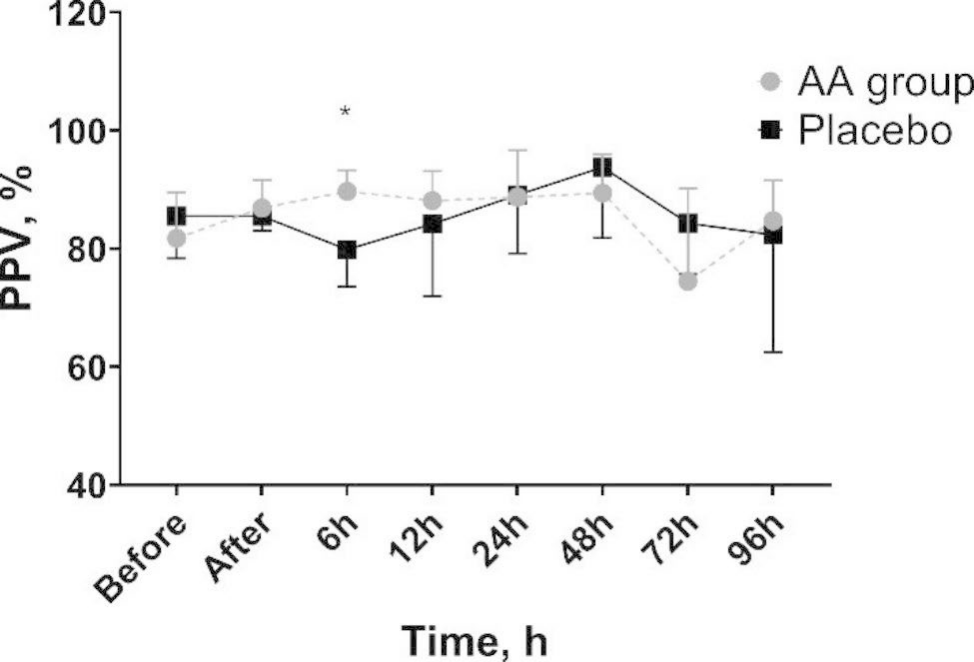
PPV changes in ascorbic acid and control groups throughout the study AA—ascorbic acid; PPV—proportion of perfused small vessels; * p < 0.05 between groups Data are presented as median and 25th and 75th percentiles

**Fig. 3:**
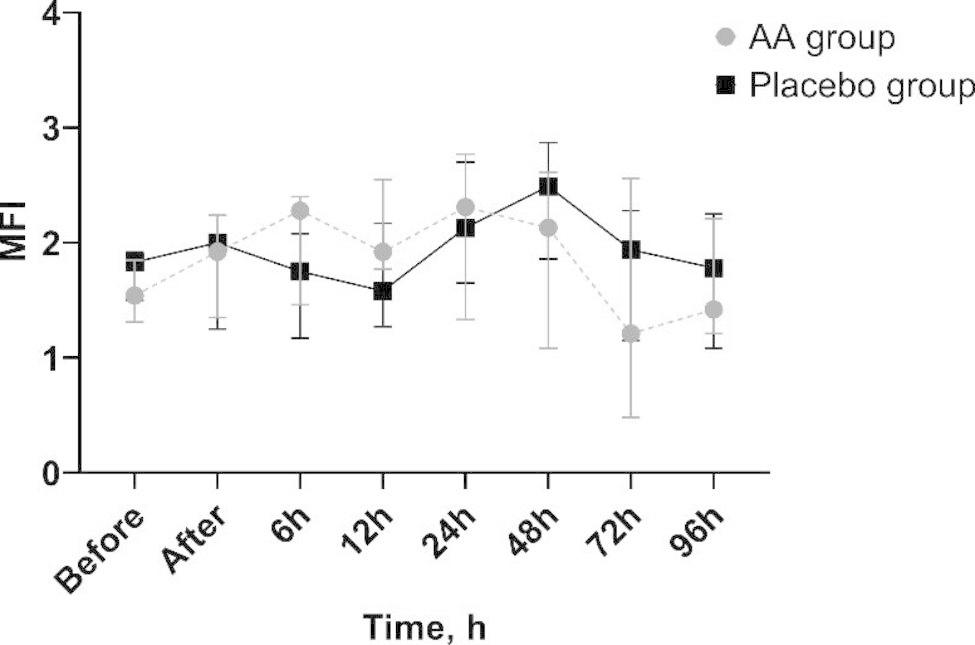
MFI changes in ascorbic acid and control groups throughout the study AA—ascorbic acid; MFI - microvascular flow index of small vessels; Data are presented as median and 25th and 75th percentiles

No differences in microcirculation flow and density parameters were found between the groups at the beginning of the study. TVD and PVD did not change significantly over the course of the study. However, PPV 6 h after the beginning of the trial was significantly higher in patients receiving ascorbic acid (89.7 (82.5–93.3) %) compared with placebo (79.9 (73.5–86.4) %, p = 0.041) (Fig. 2). There were no significant changes in MFI (Fig. 3) and FHI over the course of the study (Additional file 1).

### Evaluation of glycocalyx

**Fig. 4:**
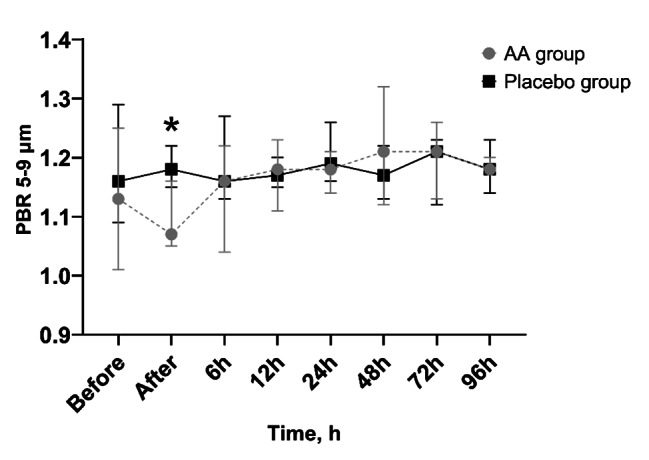
Changes of PBR in 5–9 microns diameter vessels AA—ascorbic acid; PBR - perfused boundary region; * p < 0.05 between groups Data are presented as median and 25th and 75th percentiles

No significant differences in PBRs were observed between the groups at the beginning of the study. However, a significantly lower PBR was found in the AA group in capillaries of the 5–9 μm diameter group immediately after the first ascorbic acid infusion in the AA group compared with placebo: 1.07 (1.05–1.16) μm vs. 1.18 (1.15–1.22) μm, p = 0.015 (Fig. 4). No other significant differences in PBR were observed throughout the study.

## Discussion

This study, to our knowledge, is the first to investigate the effects of ascorbic acid on microcirculation during sepsis in humans using in vivo video microscopy.

We found a statistically significantly higher proportion of perfused small blood vessels in the experimental group 6 h after the beginning of the study. No other significant differences were found between microcirculation flow and density parameters. Physiologically, there is reason to believe that ascorbic acid at sufficiently high doses should act as a vasodilator in microvessels both directly and through endothelial nitric oxide synthase (eNOS). Increased NADPH oxidase and iNOS activity, impaired mitochondrial oxidative phosphorylation, and altered eNOS expression play a vital role in the evolution of sepsis-induced endothelial dysfunction [27, 28]. Under inflammatory conditions, NADPH becomes one of the predominant reactive oxygen species (ROS) sources in endothelial cells. NADPH-originated ROS stimulates the production of iNO via iNOS expression [27]. As oxidative stress builds up in the body, ROS oxidizes the eNOS cofactor BH4, thereby reducing the activity of eNOS, which is responsible for endothelial NO synthesis. By stimulating soluble guanylate cyclase and increasing cGMP concentration in endothelial cells, eNO induces vasodilation and inhibits platelet aggregation, activates platelet, and inhibits leukocyte adhesion. It is for these reasons that eNO is necessary to maintain optimal microcirculation. In the absence of BH4, eNOS, instead of producing NO, begins to produce superoxide. When bound to NO (non-eNOS-derived), the latter forms peroxynitrite, the most dangerous and reactive free radical [28].

The role of AA in these reactions has been demonstrated in in vitro studies. AA reduces NADPH oxidase activity [27] and iNOS expression [29], increases the bioavailability of NO in endothelial cells, prevents BH4 oxidation, and allows recovery of BH4, thus reducing ROS production [30]. Recovery of BH4 helps in the appropriate maintenance of eNOS activity and endothelial-dependent NO synthesis and, hence, endothelial-dependent vasodilation [28]. The mechanisms could explain the rapid effect of ascorbic acid on microcirculation.

The importance of AA in endothelium-dependent vasodilation has also been demonstrated in preclinical animal studies. Parenteral administration of various doses (10–200 mg/kg) of vitamin C during experimental sepsis in the very early period prevents sepsis-induced microcirculatory alterations and facilitates subsequent restoration of impaired microcirculation 6 h from the onset of sepsis [2, 8, 12, 31]. After administration of 200 mg/kg of AA prior to experimental sepsis, capillary vascular reactivity was preserved, and worsening vasoconstriction was prevented [32]. In addition, this dose of AA prevented an increase in capillary permeability [33].

There are a couple of published studies on high-dose AA treatment involving humans. Friedrich Mittermayer and colleagues investigated eight healthy volunteers infused with *Escherichia coli* endotoxin [34]. The authors determined the effect of intraarterially administered AA on forearm circulation. They found that AA increases BH4 levels and completely restores the endothelium-dependent response to acetylcholine. Intraarterial infusion of vitamin C also helped to restore the circulatory response in forearm vessels to norepinephrine and angiotensin II, but blood flow at the microcirculation level was not independently evaluated in this study [35]. Given that sepsis is characterized by incoherence between systemic hemodynamics and microcirculation [7], it may not be entirely accurate to extrapolate these data to effects on microcirculation alone.

A study by Jean-Remi Lavillegrand and colleagues showed that a relatively low dose (40 mg/kg) of intravenous ascorbic acid helps to maintain vascular reactivity during sepsis, which was measured in the forearm area by transdermal iontophoresis of acetylcholine [36]. The authors also recorded an improvement in clinical signs of tissue perfusion (decreased skin mottling score, decreased capillary refill time, and decreased gradient of central and peripheral temperatures) as early as one hour after single infusion. Although only half of the patients were vitamin C deficient, such a response was observed regardless of the initial serum AA levels and presence of hypoperfusionA significant positive correlation was found between the initial AA level and endothelial response within 1 h after infusion. In our study, AA blood levels have not been evaluated, but studies by other authors suggest that it is very low in patients with sepsis and septic shock. Anitra C. Carr showed that 88% of septic patients experienced AA deficiency regardless of daily AA supplementation (100–200 mg/day) according to recommendations [10]. In other studies, all patients were AA deficient [11, 37].

It is important to mention that deficiency of ascorbic acid during sepsis is detected not only in serum but also intracellularly (24). Data on how intracellular level of AA changes during the administration of high dose of AA intravenously is lacking. It might be important for microvascular blood flow changes considering the fact that intracellular AA helps to restore endothelial function by inhibiting NADPH oxidase dependent processes. (330)

We did not find significant differences in PBRs, as a marker of glycocalyx thickness between the groups except forin the smallest diameter capillaries immediately after the first ascorbic acid infusion. Data show that recovery of the hemodynamically significant glycocalyx layer may take up to several days after various lesions [38]. However, under specific conditions, endothelial cells appear to have the potential to regenerate the glycocalyx entirely within 24 h from violation [38, 39]. One research showed glycocalyx recovery during experimental hemorrhagic shock in rats within 1 h after administration of 15 mL/kg of fresh-frozen plasma [40]. The glycocalyx’s dynamic adaptive nature or plasticity, which may allow instantaneous thinning of the permeable layer of the glycocalyx, should be considered. However, from a functional point of view, all possible modifications to the nanomechanical properties of the endothelial surface are important, and a collapsed or degraded glycocalyx may have adverse effects on the microvascular system [41]. There is evidence in the literature that a high dose of intravenous AA reduces glycocalyx damage during sepsis and ARDS [9]. In that study, however, the concentration of the glycocalyx degradation product syndecan–1, rather than the glycocalyx thickness, was assessed.

Surprisingly, we did not observe any statistically significant differences in the dose of vasopressors between groups during the study. Bearing in mind that AA is an important cofactor in noradrenaline synthesis and its deficiency is associated with decreased noradrenaline levels [42], a need for lower doses of vasopressors in the experimental group could be expected. Data in the literature on this issue are contradictory. Several studies show that AA does not affect vasopressor dose or shock duration [37, 43, 44]. Several authors report a significantly reduced duration of vasopressor infusion, an effect that is explicitly associated with ascorbic acid [45–49]. Several reasons may explain the differences. In particular, in studies in which no significant differences were observed between the dose and duration of the vasopressor infusion, a fixed dose of 6 g/day of vitamin C was administered. This dose may be too low to affect the hemodynamics. Furthermore, AA levels have only been studied in solitary studies, but data suggest that it is not always possible to increase them sufficiently, even through administering higher doses (100–200 mg/kg/day) [49, 50]. The initial dose of noradrenaline was not specified in some studies (only the duration of administration was reported) [47, 49] or was lower [42] compared with studies in which an effect on hemodynamics was observed [37, 43, 44]. The previously mentioned study by Jean-Remi Lavillegrand and colleagues could explain this discrepancy as the patients with higher initial AA levels (which could lead to a lower initial need for vasopressors) and receiving AA showed more significant improvements in their blood flow compared with those with lower initial AA levels [36].

Potentially beneficial ascorbic acid’s role was challenged by a recently published LOVIT trial [51]. In contrast to numerous previous studies, the authors of this study reported higher incidence of death and persistent organ dysfunction at 28 days in adult septic patients treated with high dose of ascorbic acid [52]. The result of this trial makes it clear that high dose of ascorbic acid should not be routinely administered for all septic patients. Sepsis is however particularly complex and heterogeneous pathology combining different mechanisms. Different clinical phenotypes of sepsis, characterized by different response to the same treatment are more and more often discussed. What is found to be effective in one phenotype, in other cases might even have deleterious effect [53] This might explain such conflicting results.

This hypothesis is supported by Sun-Young Jung and collegues [54]. The authors evaluated 36 327 patients treated with ascorbic acid and control group of the same size. It was found that AA alone and in combination with thiamine reduced mortality in patients older than 70 years with more comorbidities, for those whose predominant source of infection was pneumonia and urinary tract as well in patients with septic shock requiring mechanical ventilation [54]. In opposite there was no improvement in the mortality of the patients with abdominal sepsis and those requiring renal replacement therapy. Duration of treatment must be also taken into consideration. Interesting insights were made by Hyun Jung Lee in an experimental peritonitis study with mice. AA (45 mg/kg), thiamine and hydrocortisone was administered alongside the usual sepsis treatment for four days [55]. 50% of mice in the control group have died within 3 days meanwhile no deaths during this period occurred in the experimental group. When adjunctive treatment was terminated, animals began to die. Similar pattern was demonstrated in CITRIS - ALI study [49].

On the other hand in the context of microcirculation there are plenty of animal data indicating that even a single dose of intravenous AA might help to prevent sepsis induced microvascular alterations and restore microvascular properties [2, 8, 12, 31].

The main limitation of this study is the small sample size, but patients were recruited in the study following strict inclusion criteria within the first 24 h after ICU admission.

Abdominal sepsis was predominant in the experimental group. *E. coli* is a characteristic pathogen for sepsis of this origin, which has specific features. Experiments with primates have shown that in 15% of cases, *E. coli* causes irreversible capillary thrombosis [56]. In addition, dissociation between sublingual and intestinal microcirculation is typical during abdominal sepsis, and the sublingual response to therapeutic measures differs from the changes recorded in the intestine [57, 58]. Severe microvascular flow alterations and relatively higher FHI in our study could also be partially explained by the very severe condition of the patients in both groups with high mortality, and small sample size.

We did not determine the serum AA levels of our patients and, therefore, do not know if they were initially deficient, but many other studies have shown that AA levels are already deficient in patients in the first hours of sepsis, comparable to the levels observed in scurvy [10, 11]. We do not know how much these levels were raised by our chosen dose of AA, but we expect it should result in millimolar-level concentrations based on previous studies [11]. Though assignment was random, the study group comprised more male individuals. Bodyweight is considered to be an essential factor for gender-related differences in the pharmacokinetics of vitamin C [59]. Ascorbic acid was, however, dosed according to the patient’s weight, which did not differ significantly between groups.

Syndecan-1 levels were not reported. Syndecan-1 would allow to evaluate degree of the glycocalyx damage systemically throughout the body. Imaging device we have used allows investigating the area of interest in real-time to evaluate the dynamics and the thickness of the glycocalyx in microvessels of different diameters, which is very important in the context of microcirculation.

## Conclusions

High-dose parenteral ascorbic acid tends to increase the proportion of perfused microvessels in the early period of sepsis and septic shock. Future studies with high-dose intravenous ascorbic acid would allow elucidating whether the cause of infection may influence changes in microcirculation and address the optimal timing and duration of administration.

### Electronic supplementary material

Below is the link to the electronic supplementary material.

Supplementary Material 1

## Data Availability

The datasets used and/or analysed during the current study available from the corresponding author on reasonable request.

## Abbreviations

AA: Ascorbic acid
APACHE II: The Acute Physiology and Chronic Health Evaluation II score
ARDS: Acute respiratory distress syndrome
ATP: Adenosintriphosphate
BH4: Tetrahydrobiopterin
eNOS: Endothelial nitric oxide synthase
FHI: Flow heterogeneity index
IDF: Incident dark field
iNO: Inducible nitric oxide
iNOS: Inducible nitric oxide synthase
MFI: Microvascular flow index of small vessels
NADPH: Nicotinamid adenine dinucleotide phosphate
NO: Nitric oxide
PBR: Perfused boundary region
PPV: Proportion of perfused small vessels
PVD: Perfused small vessel density
RBC: Red blood cell
ROS: Reactive oxygen species
SDF: Sidestream dark field
SOFA: Sequential Organ Failure Assessment
TVD: Total small vessel density
