# Supplementary material for: Effect of high-dose intravenous ascorbic acid on microcirculation and endothelial glycocalyx during sepsis and septic shock: a double-blind, randomized, placebo-controlled study
Source: BMC Anesthesiol. 2023 Sep 12;23:309. doi: 10.1186/s12871-023-02265-z (PMC10496271; doi:10.1186/s12871-023-02265-z)
Supplement: Supplementary file 1 — Supplementary Material 1 [file 12871_2023_2265_MOESM1_ESM.pdf]

Additional file 1. Changes of microcirculatory parameters over the course of the study

	0h Before	0h After	6 h	12 h	24 h	48 h	72 h	96 h	P value (Friedman test)
**TVD** (mm/mm^2)									
Placebo	21.7 (18.8 - 23.1)	22.5 (21.0 - 24.1)	19.3 (18.4 – 23.8)	22.6 (19.9 – 23.4)	21.2 (19.5 – 22.5)	22.0 (19.8 – 23.1)	23.8 (22.4 – 24.9)	21.7 (21.1 – 22.9)	0.406
Vit C	21.4 (19.6 - 24.3)	19.4 (17.2 - 27.5)	22.6 (19.7 – 26.2)	22.9 (18.4 – 25.2)	23.1 (20.1 – 24.7)	21.1 (19.7 – 23.3)	23.4 (17.1 – 25.7)	24.0 (23.5 – 24.0)	0.817
**PVD** (mm/mm^2)									
Placebo	18.1 (14.4 - 21.5)	19.3 (17.5 - 21.9)	15.9 (15.4 – 19.4)	16.7 (15.2 – 20.5)	18.5 (16.9 – 19.6)	18.7 (17.0 – 22.0)	19.6 (17.5 – 21.3)	17.4 (15.5 – 19.2)	0.446
Vit C	16.7 (15.3 - 20.8)	18.3 (14.3 - 23.9)	18.9 (17.8 – 24.7)	19.7 (16.3 – 22.7)	21.4 (13.0 – 22.1)	18.9 (15.9 – 22.3)	18.3 (10.5 – 22.1)	19.5 (19.2 – 21.5)	0.907
**PPV** (%)									
Placebo	83.4 (78.4 - 90.9)	85.5 (83.0 - 90.9)	79.9 (73.5 – 86.4)*	84.2 (72.0 – 89.9)	89.1 (79.2 – 92.6)	93.8 (81.8 – 96.3)	84.3 (75.8 – 91.9)	82.3 (62.4 – 89.5)	0.579
Vit C	81.8 (69.5 - 89.6)	86.9 (77.4 - 91.6)	89.7 (82.5 – 93.3)	88.1 (84.6 – 93.1)	88.7 (69.2 – 96.6)	89.4 (81.0 – 95.9)	74.5 (59.6 – 90.2)	84.8 (81.7 – 91.5)	0.230
**MFI**									
Placebo	1.83 (1.50 - 1.92)	2.00 (1.25 - 2.07)	1.75 (1.17 – 2.08)	1.58 (1.27 – 2.17)	2.13 (1.65 – 2.70)	2.49 (1.86 – 2.87)	1.94 (1.15 – 2.28)	1.78 (1.08 – 2.25)	0.508
Vit C	1.54 (1.31 - 1.85)	1.92 (1.35 - 2.24)	2.28 (1.46 – 2.40)	1.92 (1.77 – 2.55)	2.31 (1.33 – 2.77)	2.13 (1.08 – 2.61)	1.21 (0.48 – 2.56)	1.42 (1.21 – 2.21)	0.344
**FHI** (AU)									
Placebo	0.28 (0.22-0.445)	0.20 (0.15-0.45)	0.40 (0.22-0.54)	0.18 (0.00-0.46)	0.46 (0.13-0.50)	0.29 (0.13-0.46)	0.40 (0.17-0.45)	0.30 (0.13-0.39)	0.751
Vit C	0.25 (0.15-0.36)	0.35 (0.02-0.39)	0.29 (0.11-0.38)	0.14 (0.00-0.29)	0.27 (0.10-0.43)	0.17 (0.03-0.34)	0.30 (0.27-0.42)	0.19 (0.12-0.12)	0.531

Vit C – vitamin C, TVD – total vessel density, PVD – perfused vessel density, PPV – proportion of perfussed vessels, MFI – microvascular flow index, FHI – flow heterogeneity index, * <0,05 compared with placebo group

Data are presented as median and 25th and 75th
